# Accuracy of a Symptom-Based Approach to Identify Hypertensive Emergencies in the Emergency Department

**DOI:** 10.3390/jcm9072201

**Published:** 2020-07-12

**Authors:** Fabrizio Vallelonga, Federica Carbone, Francesco Benedetto, Lorenzo Airale, Silvia Totaro, Dario Leone, Anna Astarita, Eleonora Avenatti, Simona Maule, Franco Veglio, Enrico Lupia, Alberto Milan

**Affiliations:** 1 Division of Internal Medicine and Hypertension, Department of Medical Sciences, University of Turin, Via Genova 3, 10126 Turin, Italy; lorenzo.airale@gmail.com (L.A.); astarita.unito@gmail.com (A.A.); eleonoravenatti@gmail.com (E.A.); simona.maule@gmail.com (S.M.); franco.veglio@unito.it (F.V.); alberto.milan@gmail.com (A.M.); 2 Division of Emergency Medicine, AOU Città Salute e Scienza, Torino, Department of Medical Sciences, University of Turin, 10126 Turin, Italy; federica.carbone8@gmail.com (F.C.); francesco.benedetto@edu.unito.it (F.B.); s.totaro84@gmail.com (S.T.); dgleone@live.it (D.L.); enrico.lupia@unito.it (E.L.)

**Keywords:** hypertensive emergencies, diagnostic approach, symptoms accuracy

## Abstract

Background: A hierarchical symptoms-based diagnostic strategy relying on the presence of five main symptoms (chest pain, acute dyspnea, neurological symptoms, headache, visual impairment) was recently proposed to diagnose patients with hypertensive emergency. However, poor scientific evidence is available about the role of symptoms in both diagnosis and management of acute hypertensive disorders. Methods: Data from 718 patients presenting to the emergency department of the “Città della Salute e della Scienza” Hospital of Turin with systolic blood pressure > 180 and/or diastolic blood pressure > 110 mm/Hg were retrospectively analyzed. The accuracy of the typical symptoms for identification of hypertensive emergencies was assessed. Results: A total of 79 (11%) out of 718 patients were diagnosed with hypertensive emergencies (51% had cardiovascular and 49% neurovascular acute organ damage). Patients with hypertensive emergencies were older and with higher prevalence of coronary artery disease and chronic heart failure than patients with uncontrolled hypertension. Typical symptoms could discriminate true hypertensive emergency from uncontrolled hypertension with 64% accuracy, 94% sensitivity, and 60% specificity. Conclusion: Typical symptoms might be used as a simple screening test (99% negative predictive value) in the emergency department to select for further evaluations of patients with suspected hypertensive emergencies among those with acute hypertensive disorders.

## 1. Introduction

Hypertensive emergencies are characterized by an acute and severe blood pressure (BP) rise (>180/110 mm/Hg) with associated acute hypertension-mediated target organ damage (aHMOD). The term “hypertensive urgency”, previously adopted to define acute BP rise without aHMOD, is currently discouraged as there is no evidence of different prognosis nor need of different management for these patients compared to outpatients with asymptomatic uncontrolled hypertension [1]. True hypertensive emergencies are rare, but their prevalence has more than doubled in the United States in the last few years [2,3], while in Italian emergency departments (EDs), acute hypertensive disorders showed only a minimal reduction in prevalence from 2008 to 2015 (from 2% to 1.76% of all admissions) [4], despite significant improvement in hypertension management.

Limited scientific evidence is currently available on the role of symptoms in both diagnosis and management of acute hypertensive disorders. A hierarchical strategy relying on the presence of five main symptoms (chest pain, acute dyspnea, neurological symptoms, headache, visual impairment) was recently proposed to screen patients with suspected hypertensive emergencies [1], but the accuracy of this symptom-based approach has not been validated.

The aim of our study is to assess the accuracy of a symptoms-based diagnostic strategy in distinguishing patients with hypertensive emergencies from those with severe uncontrolled hypertension.

## 2. Methods

We reviewed clinical records of all patients admitted to the ED of the “Città della Salute e della Scienza” Hospital of Turin, from 1 January 2018 to 30 June 2018. Patients were included in the study as per the following inclusion/exclusion criteria:

### 2.1. Inclusion Criteria

Presentation to the ED with systolic BP > 180 mm/Hg and/or diastolic BP > 110 mm/Hg; BP values had to be observed at first assessment by nursing staff and confirmed at medical evaluation.

### 2.2. Exclusion Criteria

High BP at first assessment by nursing staff, not confirmed at medical evaluation; high BP due to traumatic causes or known neoplastic pain; incomplete anamnestic and clinical data.

## 3. Medical Record Review and Definitions

The following data were collected from medical records (ED report and electronic hospital database): past medical history, ongoing cardiovascular therapy, acute presenting symptoms, vital signs on ED admission, biochemical data during ED evaluation.

### 3.1. Past Medical History

The following data were collected:

- Cardiovascular risk factors: smoking habit, history of arterial hypertension, diabetes mellitus, dyslipidemia;
- Previous cardiovascular events: coronary artery disease, chronic heart failure, atrial fibrillation (paroxysmal, persistent, and permanent), chronic kidney disease (i.e., glomerular filtration rate < 60 mL/min/1.73 m^2^), previous ischemic or hemorrhagic stroke, previous transient ischemic attack.

### 3.2. Ongoing Cardiovascular Therapy

Data on antihypertensive drugs (angiotensin converting enzyme inhibitors, angiotensin II receptor antagonists, diuretics, calcium channel blockers, beta blockers, alpha blockers, alpha−2 agonists), antiplatelets and anticoagulation therapy (vitamin K antagonists, direct oral anticoagulants) were collected, as outlined before.

### 3.3. Presenting Symptoms

The symptoms reported by patients on ED admission were recorded, according to the following clusters:

- Main symptoms: chest pain (both typical and atypical), dyspnea (as subjective perception), neurological focal signs (motor, sensory, or speech symptoms), headache (regardless of severity), visual impairment (reduced visual acuity or visual fields).
- Less specific symptoms: vertigo, confusion, epistaxis, syncope or pre-syncope, nausea or vomiting, abdominal pain.
- Unspecific symptoms: all other reported symptoms.

More than one symptom per patient was recorded when necessary.

### 3.4. Vital Signs on Admission

**Figure 1:**
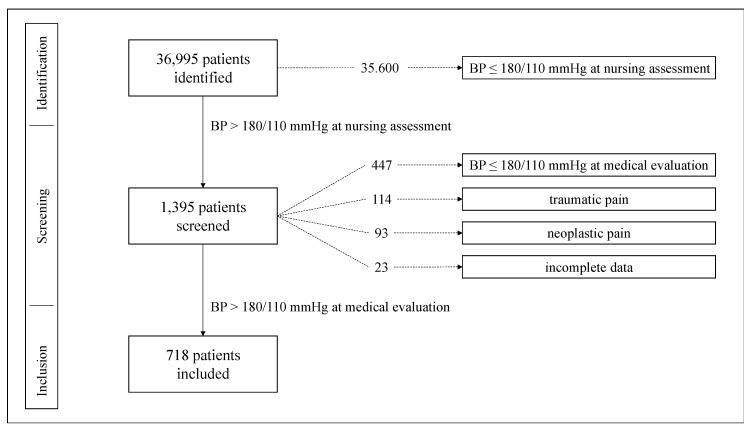
Flowchart of patient selection process. BP: blood pressure.

BP values assessed by nursing staff and at subsequent medical evaluation were both recorded. BP assessed by nursing staff was used as the main criterion during the patient’s selection process (Figure 1); BP during medical evaluation was considered the admission BP and is reported in Table 1. Heart rate and oxygen saturation were gathered from medical evaluation.

### 3.5. Laboratory Analysis

The following biochemical data were collected whenever available: hemoglobin, creatinine, sodium, potassium, troponin T, *n*-terminal pro-hormone brain natriuretic peptide, lactate dehydrogenase.

### 3.6. Hypertensive Emergency and Uncontrolled Hypertension

Acute coronary syndrome, acute heart failure, aortic dissection, ischemic or hemorrhagic stroke, transient ischemic attack, hypertensive encephalopathy, malignant hypertension with or without thrombotic microangiopathy, and progressive renal failure were considered aHMOD, according to current recommendations [1]. Briefly, malignant hypertension has been defined in the presence of hypertension-multiorgan damage [5]. Acute renal failure was defined as an increase in serum creatinine ≥1.5 times compared to baseline value [6], as reported in the electronic hospital database. Final diagnoses, and thus aHMOD, were assigned by two independent researchers through review of full ED reports; any discordances between them were resolved by a third, blinded, researcher.

Patients were divided in two groups, according to the following criteria [1]:

- Hypertensive emergency: systolic BP > 180 mm/Hg and/or diastolic BP > 110 mm/Hg and associated aHMOD;
- Uncontrolled hypertension: systolic BP > 180 mm/Hg and/or diastolic BP > 110 mm/Hg without aHMOD.

The local institutional review board (Comitato Etico Interaziendale Città della Salute e della Scienza di Torino, Torino, Italy) approved the study and all participants provided informed consent (Protocol Number, Practice Number /2019).

## 4. Statistical Analysis

Normal distribution of variables was tested using the Shapiro-Wilk and residual analysis tests. Continuous variables were expressed as mean ± standard deviation. Qualitative variables were expressed as absolute values of frequency and percentage values. Differences between independent groups were evaluated using a t-test for continuous variables with normal distribution and the Mann–Whitney or Kruskal–Wallis test for continuous variables with non-normal distribution. Categorical variables were compared using the chi-square test or Fisher’s exact test, as appropriate. A binary logistic regression was applied, using the presence of hypertensive emergency as a dependent variable, and the statistically different variables between patients with hypertensive emergency and those with uncontrolled hypertension, as independent variables. Statistical significance was considered for *p* values < 0.05 in all analysis. Statistical analysis was performed with software package SPSS (Statistical Package for the Social Sciences–version 22–© 2020 IBM).

### Symptoms Accuracy

Minimal sample size required to detail the accuracy of the symptoms-based diagnostic strategy was calculated based on the hypothesized values of sensitivity and specificity of this approach and on the prevalence of hypertensive emergencies in our population (almost 10%). Assuming a sensitivity of 90% and a specificity of 50%, with 95% confidence intervals width of 10%, the sample size required was of 346 for the expected sensitivity and 107 for the expected specificity [7]. Since there was no need for independent validation, we determined accuracy over the entire population to obtain a more reliable result.

A 2 × 2 contingency table was built using the presence/absence of main symptoms as diagnostic test and the presence/absence of hypertensive emergency as real outcome. The absence of a relationship between main symptoms and hypertensive emergency was the null hypothesis to be rejected; the chi-square test was used to assess for independence between two conditions. The same 2 × 2 contingency table was used to derive the following measures of diagnostic accuracy: sensitivity, specificity, positive likelihood ratio, negative likelihood ratio, positive predictive value (PPV), and negative predictive value (NPV).

## 5. Results

Out of 36,995 patients consecutively admitted to our ED during the study period, 718 (1.9% of all admissions) met the inclusion criteria (Figure 1; Table 1); of those, 401 were women (56%) and 317 (44%) men. A total of 79 (11%) patients in our population were diagnosed with hypertensive emergencies: 28 (35%) had acute heart failure, 17 (22%) had transitory ischemic attack, 14 (18%) had ischemic stroke, 12 (15%) had acute coronary syndrome, 7 (9%) had hemorrhagic stroke, and 1 (1%) had acute aortic dissection. No diagnosis of hypertensive encephalopathy, malignant hypertension or hypertension-mediated acute renal failure was assigned.

Patients with hypertensive emergencies were older (73.2 ± 13 vs. 69.5 ± 14 years, *p* = 0.03) and had higher prevalence of coronary artery disease (24% vs. 11%, *p* < 0.01) and chronic heart failure (6% vs. 2%, *p* = 0.04) compared to those with uncontrolled hypertension. No differences in sex prevalence (women 54% vs. 56% emergencies and uncontrolled hypertension respectively, *p* = 0.79), known arterial hypertension (77% vs. 78%, *p* = 0.81), and other cardiovascular risk factors were found between the two groups. Moreover, a similar percentage of patients in both groups was on antihypertensive therapy (72% vs. 67%, *p* = 0.44), but a higher proportion of patients with hypertensive emergencies was chronically taking three or more antihypertensive drugs (32% vs. 21%, *p* = 0.03). A more frequent use of beta-blockers and loop diuretics, as well as acetylsalicylic acid, was observed in the hypertensive emergencies group (Table 2). Among patients with known arterial hypertension, 16% in both groups reported not to be on active treatment, although they had active prescriptions of antihypertensive medications by their primary care physician.

A higher concentration of serum Troponin T (75.3 ± 133 ng/L vs. 23.1 ± 49 ng/L, *p* = 0.02—normal value < 30 ng/L) was noted in patients with hypertensive emergencies; no further differences were identified comparing the other main biochemical data (Table 3).

The most represented variables in the aHMOD group, such as previous coronary artery disease or chronic heart failure, the use of ≥3 antihypertensive drugs, age, and the presence of at least one main symptom were all associated with hypertensive emergencies on univariate logistic regression, but only the last two variables retained their significance on multivariate analysis (Table 4). Notably, presence of main symptoms showed the strongest association (OR 18.314, 95% CI 7.82–42.9, *p* < 0.01).

### Symptoms-Based Diagnostic Strategy

Presenting symptoms are reported in Table 5. Patients with hypertensive emergencies more frequently had dyspnea (32% vs. 11%, *p* < 0.01) and neurological focal signs (38% vs. 5%, *p* < 0.01) compared to patients with uncontrolled hypertension; on the other hand dizziness, epistaxis, syncope or pre-syncope, palpitations, and other less specific symptoms were almost exclusively present in the latter group of patients.

The vast majority (94%; 74 out of 79) of patients with hypertensive emergencies had at least one main symptom; in the 5 out of 79 with less specific symptoms, one patient reported abdominal pain (final diagnosis: aortic dissection), one patient reported vertigo (final diagnosis: transient ischemic attack), one patient presented with palpitations, and two with abdominal discomfort (final diagnosis: acute heart failure for all three). No differences in age, gender, cardiovascular comorbidities were found between patients with main symptoms and those with less specific symptoms in the hypertensive emergency’s cohort.

**Figure 2:**
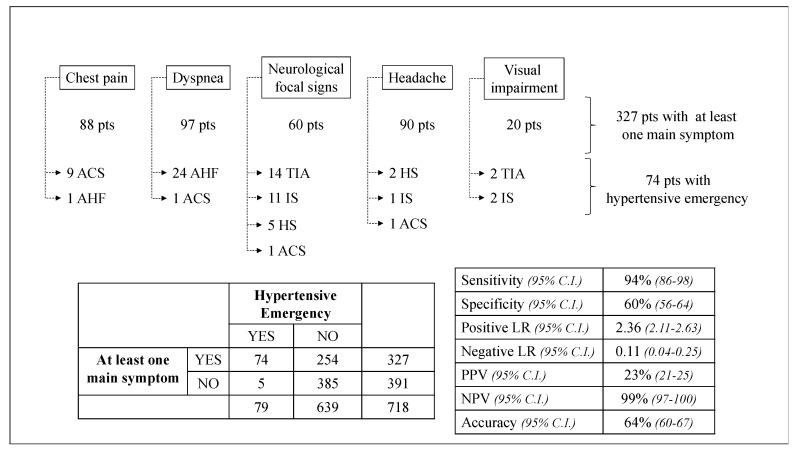
Accuracy of the symptoms-based diagnostic strategy as screening assessment. ACS: acute coronary syndrome; AHF: acute heart failure; TIA: transient ischemic attack; IS: ischemic stroke; HS: hemorrhagic stroke; pts: patients; LR: likelihood ratio; PPV: positive predictive value; NPV: negative predictive value.

No patient with a hypertensive emergency was asymptomatic or only reported totally unspecific symptoms. In our study population, the accuracy of the symptom-based diagnostic strategy, proposed by Van den Borg and colleagues [1] for the identification of patients with true hypertensive emergencies among those with acute hypertension, was 64%, with a sensitivity of 94%, a specificity of 60%, a NPV of 99% and a PPV of 23% (Figure 2). The symptom-specific PPV was 11.3% for the presence of chest pain, 25.8% for dyspnea, 51.6% for focal neurological signs, 4.4% for headache, and 20% for visual impairment.

## 6. Discussion

This study evaluated the accuracy of the main emergency symptoms in the detection of patients with hypertensive emergencies among those with acute hypertensive disorders. The recently proposed symptom-based diagnostic strategy [1] showed 64% accuracy, 94% sensitivity, 60% specificity, 99% NPV, and 23% PPV in our cohort. To our knowledge, limited scientific evidence is currently available about the role of symptoms in both diagnosis and management of acute hypertensive disorders; moreover, no validation data regarding the diagnostic strategy proposed by the expert consensus have been provided.

Hypertensive emergencies are rare but serious medical conditions, accounting for about 2‰ of total ED admission and 10% of patients with acute hypertensive disorders in our retrospective analysis, in line with previously published data [4,8,9,10]. Clinically, the main features of hypertensive emergencies are chest pain, dyspnea, focal neurological symptoms, headache, and visual impairment; symptoms other than these are less frequent and not associated with aHMOD. Recently a symptom-based diagnostic strategy to identify hypertensive emergency was proposed by Van den Borg and colleagues [1]; when applied to our patient population, this diagnostic approach showed a great sensitivity (94%) in the identification of patients with hypertensive emergencies.

In our cohort of hypertensive emergencies, 49% presented with a cerebrovascular event (ischemic and hemorrhagic in about 80% and 20%, respectively), 35% with acute heart failure, 15% with acute coronary syndrome, and 1% with acute aortic dissection, distributions that are in line with recently reported data from different populations [4,11,12]. No cases of hypertensive encephalopathy emerged from our analysis, despite a prevalence between 5% [13] and 18% [14] reported in past studies. This finding could reflect how challenging this diagnosis is in ED, due to the low sensitivity of head computed tomography (CT) in detecting cerebral edema, compared to the less frequently used magnetic resonance imaging (MRI) or even thorough clinical assessment with fundoscopic evaluation [15]. In this regard, among 157 patients with neurological signs, headache, or visual impairment in our cohort, only three brain MRI and two fundoscopic examinations were performed. Furthermore, no cases of malignant hypertension were detected in our population. Once again this data may be due to diagnostic difficulties in an emergency setting [16], as well as to the relatively low prevalence of the disease, reported having an annual incidence of 2 per 100,000 in the Caucasian population [17].

Patients with hypertensive emergencies were older than patients with severe uncontrolled hypertension and the mean age of our population was in line with the most recent Italian studies [4,13]. About a quarter of the patients included in our study had no previous history of hypertension; a high percentage that has, however, been previously reported [4,13,14]. Considering only patients with known hypertension, 16% declared non-adherence to antihypertensive treatment, as described in a targeted analysis [18].

Patients with hypertensive emergencies had higher prevalence of coronary artery disease and chronic heart failure compared to uncontrolled hypertension in our population, once again in line with previous reports [4,19]. This finding may explain the greater use of beta-blockers, loop diuretics and acetylsalicylic acid in the former group. Curiously, none of the considered cardiovascular risk factors (smoking habit, arterial hypertension, diabetes mellitus, dyslipidemia) was associated with hypertensive emergencies; this surely does not discredit the role of cardiovascular risk factors in patients’ management, but underlines the need for a risk model stratification specifically designed for acute hypertensive disorders, as only recently proposed [20,21]. Indeed, although the importance of traditional risk factors on long-term cardiovascular risk has been confirmed [22], the ability of these parameters to predict, for example, acute coronary syndrome resulted poorly in ED patients > 40 years old [23]. In younger patients only the presence of >4 risk factors helped to predict myocardial infarction [24].

## 7. Symptoms Accuracy and Clinical Implications

Chest pain, dyspnea, and neurological focal signs were the most frequent symptoms in hypertensive emergencies in our population, in line with previous reports [4,11,14], although headache was equally common in other reports [8]. Logistic regression analysis showed a strong association between main symptoms and hypertensive emergencies (OR 18.314, 95% CI 7.82–42.9, *p* < 0.01), after correction for age, presence of coronary artery disease and chronic heart failure, and number of antihypertensive drugs.

We tested for the first time the association between symptoms and hypertensive emergencies, evaluating the accuracy of the recently hierarchical strategy proposed by Van den Borg and colleagues [1]. The absence of all five symptoms might rule out a hypertensive emergency with a NPV of 99% in our population, proving to be a good and simple screening method for acute hypertensive disorders. On the other hand, although we observed the presence of at least one of the main symptoms (chest pain, dyspnea, neurological focal signs, headache, visual impairment) in a significant proportion of patients with hypertensive emergencies (94%), the low PPV of the symptom-based strategy (23%) underlines the need for further tools of risk stratification to guide acute clinical management in the ED.

## 8. Limitations

The data presented are the results of a single-center analysis, although similarities with previous published data are promising indicators of a generalizability of the results. The retrospective nature and the ED setting of the present study intrinsically bring some more limitations: clinical data might not have been systematically collected at the time of the medical encounter, resulting in missing data, and BP values might not have always been acquired following updated guidelines. It has not been possible to accurately collect important data such as the precise dosage of medications or the effective adherence to the prescribed antihypertensive drugs, evaluated through the use of validated scales or therapeutic drug monitoring strategies. However, we strived to perform the most complete review possible; in order to maximize accuracy of BP values, both nurse- and physician-recorded data were used; in order to optimize the quality of clinical data both ED report and electronic hospital database were carefully examined by two independent researchers. We also considered hypertensive emergencies as a single entity in testing the accuracy of the symptoms-based diagnostic strategy; it would be interesting to perform similar analyses targeted on a single aHMOD and a specific symptom, increasing the sample size. It may also be useful to refine the symptom-based screening strategy, adding important symptom-related features, such as chest pain patterns and objective data on respiratory disorders (i.e., paO2 at arterial blood gas analysis).

## 9. Conclusions

Acute hypertensive disorders are rare but serious clinical pictures in the ED. Emergency symptoms (chest pain, dyspnea, focal neurological signs, headache, and visual impairment) are an excellent tool to rule out suspected hypertensive emergencies (NPV 99%) among patients with acute hypertensive disorders, burdened though by a low PPV.

## Figures and Tables

**Table 1:** Demographic and clinical characteristics.

	Hypertensive Emergencies (*n* = 79)	Uncontrolled Hypertension (*n* = 639)	*p*-Value
**Demographic Characteristics**			
Age (years)	73.2 ± 13	69.5 ± 14	0.03
Female sex [*n* (%)]	43 (54)	358 (56)	0.79
**Cardiovascular Risk Factors**			
Current smokers [*n* (%)]	18 (23)	138 (22)	0.81
Arterial hypertension [*n* (%)]	61 (77)	501 (78)	0.81
Diabetes mellitus [*n* (%)]	16 (20)	108 (17)	0.46
Dyslipidemia [*n* (%)]	14 (18)	88 (14)	0.34
No risk factors [*n* (%)]	11 (14)	95 (15)	0.82
**Previous Cardiovascular Events**			
Coronary artery disease [*n* (%)]	19 (24)	70 (11)	<0.01
Chronic heart failure [*n* (%)]	5 (6)	15 (2)	0.04
Atrial fibrillation [*n* (%)]	11 (14)	61 (10)	0.22
Chronic kidney disease [*n* (%)]	8 (10)	42 (7)	0.24
Previous stroke [*n* (%)]	9 (11)	40 (6)	0.09
No previous events [*n* (%)]	41 (52)	462 (72)	<0.01
**Vital Signs in ED (Medical Evaluation)**			
SBP at admission (mm/Hg)	193 ± 18	188 ± 16	0.06
DBP at admission (mm/Hg)	100 ± 16	98 ± 13	0.33
SBP at discharge (mm/Hg)	155 ± 21	151 ± 17	0.09
DBP at discharge (mm/Hg)	87 ± 12	84 ± 11	0.06
HR at admission (bpm)	88 ± 22	84 ± 18	0.23
Oxygen saturation at admission (%)	95 ± 4	96 ± 5	0.11

ED: emergency department; SBP: systolic blood pressure; DBP: diastolic blood pressure; HR: heart rate; bpm: beat per minute.

**Table 2:** Cardiovascular therapy.

	Hypertensive Emergencies (*n* = 79)	Uncontrolled Hypertension (*n* = 639)	*p*-Value
**Ongoing Antihypertensive Treatment**			
No antihypertensive drugs [*n* (%)]	22 (28)	208 (33)	0.44
1 antihypertensive drug [*n* (%)]	14 (18)	149 (23)	0.26
2 antihypertensive drugs [*n* (%)]	18 (23)	147 (23)	0.97
≥3 antihypertensive drugs [*n* (%)]	25 (32)	135 (21)	0.03
**Specific Pharmacological Classes of Antihypertensive Treatment**			
ACE-I [*n* (%)]	19 (24)	151 (24)	0.93
ARBs [*n* (%)]	17 (22)	146 (23)	0.79
Beta-Blockers [*n* (%)]	38 (48)	201 (32)	<0.01
Calcium Channel Blockers [*n* (%)]	19 (24)	132 (21)	0.49
Thiazide diuretics [*n* (%)]	6 (8)	94 (15)	0.09
Loop diuretics [*n* (%)]	21 (27)	75 (12)	<0.01
Potassium-sparing diuretics [*n* (%)]	4 (5)	30 (5)	0.88
Alpha-Blockers [*n* (%)]	5 (6)	57 (9)	0.44
Alpha−2 agonists [*n* (%)]	1 (1)	11 (2)	0.77
**Ongoing Antiplatelet and Anticoagulant Treatment**			
Acetylsalicylic acid [*n* (%)]	22 (28)	108 (17)	0.02
Vitamin K antagonist [*n* (%)]	8 (10)	36 (6)	0.12
Direct oral anticoagulants [*n* (%)]	9 (11)	45 (7)	0.17

ACE-I: Angiotensin-converting enzyme inhibitors; ARBs: Angiotensin II receptor blockers.

**Table 3:** Biochemical data.

	Hypertensive Emergencies (*n* = 79)	Uncontrolled Hypertension (*n* = 639)	*p*-Value
**Laboratory Data**			
Hemoglobin (g/dL)	*n* = 79 13.4 ± 1.9	*n* = 552 13.5 ± 1.9	0.86
Creatinine (mg/dL)	*n* = 79 1.16 ± 0.8	*n* = 546 1.08 ± 0.9	0.47
Sodium (mEq/L)	*n* = 79 139 ± 3.1	*n* = 546 139 ± 3.9	0.87
Potassium (mEq/L)	*n* = 79 4.1 ± 0.5	*n* = 79 4 ± 0.5	0.76
Troponin T (ng/L)	*n* = 43 75.3 ± 133	*n* = 268 23.1 ± 49	0.02
NTproBNP (pg/mL)	*n* = 24 7.573 ± 18.649	*n* = 86 3.513 ± 10.415	0.16
LDH (IU/L)	*n* = 20 506 ± 43	*n* = 112 483 ± 41	0.22

NTproBNP: *n*-terminal pro-hormone brain natriuretic peptide; LDH: lactate dehydrogenase; IU: international unit.

**Table 4:** Logistic regression analysis of potential predictors.

Univariate Logistic Regression Analysis				
Potential Predictors	*β* Coefficient	95% CI	Std. Error	*p*-Value
Age (years)	1.023	1.01–1.04	0.01	0.02
Coronary artery disease	2.574	1.45–4.56	0.29	<0.01
Chronic heart failure	2.811	1.01–7.96	0.53	0.04
≥3 antihypertensive drugs	1.728	1.04–2.88	0.26	0.04
Main symptoms (≥1)	18.442	7.90–43	0.43	<0.01
**Multivariate Logistic Regression Analysis**				
**Potential Predictors**	***β* Coefficient**	**95% CI**	**Std. Error**	***p*-Value**
Age (years)	1.021	1.01–1.04	0.01	0.04
Coronary artery disease	1.917	0.99–3.70	0.34	0.06
Chronic heart failure	1.693	0.50–5.72	0.62	0.40
≥3 antihypertensive drugs	1.150	0.64–2.07	0.30	0.64
Main symptoms (≥1)	18.314	7.82–42.9	0.43	<0.01

C.I.: confidence interval; Std Error: standard error.

**Table 5:** Presenting symptoms.

	Hypertensive Emergencies (*n* = 79)	Uncontrolled Hypertension (*n* = 639)	*p*-Value
**Main symptoms**			
Chest pain [*n* (%)]	13 (17)	75 (12)	0.23
Dyspnea [*n* (%)]	25 (32)	72 (11)	<0.01
Neurological focal signs [*n* (%)]	30 (38)	30 (5)	<0.01
Headache [*n* (%)]	6 (8)	84 (13)	0.16
Visual impairment [*n* (%)]	4 (5)	16 (3)	0.19
**Less specific symptoms**			
Vertigo/Dizziness [*n* (%)]	2 (3)	61 (10)	0.04
Confusion [*n* (%)]	10 (13)	61 (10)	0.38
Epistaxis [*n* (%)]	0 (0)	22 (3)	0.09
Syncope/pre-syncope [*n* (%)]	0 (0)	25 (4)	0.07
Nausea/vomiting [*n* (%)]	4 (5)	49 (8)	0.40
Palpitations [*n* (%)]	3 (4)	48 (8)	0.23
Abdominal pain [*n* (%)]	7 (9)	69 (11)	0.60
**Totally unspecific symptoms**			
Miscellaneous [*n* (%)]	0 (0)	133 (21)	<0.01
No symptoms [*n* (%)]	0 (0)	63 (10)	<0.01
